# OP28 Digi-HTA: The Assessment Method For Digital Health Technologies In Finland

**DOI:** 10.1017/S0266462324000916

**Published:** 2025-01-07

**Authors:** Jari Haverinen, Petra Falkenbach, Miia Turpeinen, Juha Röning, Jarmo Reponen

## Abstract

**Introduction:**

The ever-increasing number of new and innovative digital health technologies (DHTs) also sets new demands on health technology assessment (HTA) methods in addition to traditional HTA domains. In 2018, the Finnish Ministry of Social Affairs and Health recognized the need for new HTA methods for DHTs in Finland and commissioned method development.

**Methods:**

The development work of the new HTA method for DHTs and the findings related to it were based on three substudies:

(i) The new HTA method was developed through a literature review, expert interviews, and four multiprofessional workshops.

(ii) Feedback about new HTA recommendations was collected from healthcare decision-makers through a web-based survey (n=24). Feedback on the developed HTA framework was collected through a web-based survey for companies offering DHT products (n=8).

(iii) Initial experiences about the state of data security and protection of assessed products were gathered through the assessment process.

**Results:**

A new Digi-HTA method that supports a wide range of DHTs, such as health apps, AgeTech, artificial intelligence, and robotic solutions, was published in 2019. According to the healthcare decision-makers participating in the study, although the Digi-HTA recommendations included clear and beneficial information, their integration into healthcare decision-making processes should be improved. Responses from companies offering different DHTs indicated that the Digi-HTA framework would be an appropriate tool for performing assessments for their products. During the assessments, deficiencies in compliance with the best practices of data security and protection as well as data security problems were found.

**Conclusions:**

The rapid development of DHTs requires that the HTA methods also adapt to the development so that no new and innovative products are excluded from the assessments. In addition to the value of DHTs, their quality, such as data security and protection, should be assessed so that decision-makers can be supported in the best possible ways.

